# Laryngeal dystonia and vocal tremor response to botulinum toxin injection

**DOI:** 10.1007/s00405-024-09111-z

**Published:** 2024-12-07

**Authors:** João Viana Pinto, Isabel García López

**Affiliations:** Department of Otolaryngology Head & Neck Surgery, La Paz University Hospital, Madrid, Spain

**Keywords:** Laryngeal dystonia, Dystonic tremor, Vocal tremor, Botulinum toxin injections

## Abstract

**Purpose:**

The main objective of this study was to compare laryngeal dystonia (LD) and vocal tremor’s (VT) response to botulinum toxin injection.

**Methods:**

Retrospective study including every patient with LD or VT injected with botulinum toxin guided by electromyography, from January 1, 2010, to September 30, 2022, at a tertiary hospital centre. Improvement was assessed with the VHI-10, grade of dysphonia in a visual analogue scale (VAS; 0–10), GRBAS(I) scale (0–3) and maximum phonation time (MPT).

**Results:**

A total of 77 patients were included, 44 patients with LD and 33 with VT. There were no differences between groups on pre-treatment VHI-10, grade of dysphonia in the VAS, MPT and G, R, B, A and I at diagnosis (*p* > 0.05). S was significantly higher in patients with LD (*p* < 0.001). After the first injection, both groups showed an increase in the grade of dysphonia on the VAS and a decrease in VHI-10, G, S and I (*p* < 0.05), with a higher variation in the VAS and S parameters in the LD group compared to VT (*p* < 0.05). In the 54 patients that performed two or more injections, G, S and I had a higher decrease in patients with LD when compared to patients with VT (*p* < 0.05).

**Conclusion:**

BTX injection was successful in improving the VHI-10, grade of dysphonia on the VAS and G, S and I in the GRBAS-I scale for both DT and VT. LD seems to have a better response to BTX in comparison to VT.

## Introduction

Laryngeal dystonia (LD) manifests as arrhythmic and involuntary action-induced contractions of the laryngeal muscles [1]. The most prevalent manifestation of the disease is primary focal LD, formerly known as “spasmodic dysphonia” [2]. However, LD may be a part of a generalized neurological disorder in up to 30% of the cases [3]. LD is further classified based on the affected muscle groups. The most frequent type of dystonia, accounting for 80% of the cases, is adductor LD, which affects the thyroarytenoid (TA) and lateral cricoarytenoid (LCA) adductor muscle complex [4]. Furthermore, abductor LD, is characterized by dystonia of the posterior cricoarytenoid (PCA) muscles and mixed LD presents as involuntary contractions of both adductor and abductor muscles during phonation [5]. Less common variations of LD include breathing or singing LD [6, 7]. Since there are no oral medications with proven efficacy to treat LD [8], the mainstay of treatment is botulinum toxin (BTX) injections [9].

Vocal tremor (VT) is defined by rhythmic and involuntary oscillations of the laryngeal muscles [10] typically within the range of 4–11 Hz [11]. In the majority of cases, VT is associated with other neurological disorders, such as essential tremor, Parkinson’s disease or LD [12]. Additionally, tremor can also be present in many extra-laryngeal structures, including the base of tongue, pharyngeal walls, soft palate, and may even be associated with tremors in the hands or head [3]. VT may present as a horizontal pattern of tremor mainly attributed to laryngeal intrinsic muscles tremor, a vertical tremor resulting from contractions of the strap muscles or a mixed pattern of both horizontal and vertical contractions [13]. Treatment options for VT are many times directed to the underlying pathology. For example, essential tremor may be managed with propranolol or primidone while Parkinson’s disease may be treated with levodopa [12]. As these medications don’t seem to be very effective for laryngeal tremor [14, 15], many patients are treated with BTX injections [16].

The main objective of this study is to assess and compare the response to BTX injection treatment in patients with LD and VT.

## Methods

### Subjects

Retrospective cohort study performed from from January 1, 2010, to September 30, 2022 in the Otorhinolaryngology Department of a tertiary hospital center (Hospital Universitario La Paz). Every patient with LD or VT submitted to BTX injection were included. Patients were excluded when there was insufficient data to report results or the diagnosis wasn’t clear. Patients with abductor laryngeal dystonia were also excluded since BTX injections in these cases are performed in the PCA muscle and not in the TA muscle.

Differential diagnosis was based on medical history, voice evaluation, laryngeal examination and laryngeal electromyography (LEMG). Every patient was previously evaluated by a neurologist and complete screening tests were performed in order to assess possible secondary etiologies. When there were doubts regarding the diagnosis, patients were discussed in a multidisciplinary reunion including experienced laryngologists, neurologists and neurophysiologists. The protocol that was used for the differential diagnosis was previously discussed and reported in another study by our group [3]. Patients were classified as having LD, dystonic tremor or VT. Patients that presented with dystonia and no tremor were included in the LD group while patients with dystonic tremor or isolated tremor were included in the VT group.

### Data collection and outcomes

Data was collected by analyzing patients’ digital data. Demographic data (age at diagnosis, gender, symptom duration in months), clinical characteristics of dystonia or tremor and the neurological diagnosis were registered.

The assessed outcomes included the impact of dysphonia measured using the “Voice Handicap Index– 10” (VHI-10) questionnaire, previously validated in the Spanish language [17]. Additionally, self-reported grade of dysphonia severity was determined using a visual analogue scale (VAS) ranging from 0 (indicating “the worst voice possible”) to 10 (indicating a “perfect voice”). A perceptual evaluation was conducted using the Grade, Roughness, Breathiness, Asthenia, Strain, and Instability (GRBAS-I) scale, graded from 0 to 3. An aerodynamic assessment was carried out by measuring the maximum phonation time (MPT) in seconds.

These evaluations were performed before the first injection, one month after the initial injection, and one month following the injection that yielded the best results (for patients who received two or more injections). The best injection was defined as the one that produced superior results with acceptable side effects and was subsequently repeated with the same dosage in subsequent injections. Any side effects occurring after each injection were also recorded. The total number of injections, the doses of the injection with the best result, injection intervals and drop-out rate were also recorded.

### Clinical procedure

BTX type A was diluted with sterile saline at 2.5U/0.1 mL prior to injection. Injections were performed by an experienced laryngologist under LEMG guidance with the help of an experienced neurophysiologist.

A 27-gauge hollow polytetrafluoroethylene-coated monopolar needle was used for the injection. Needle insertion in the TA was via the cricothyroid membrane. This approach was previously described by the laryngeal electromyographic guideline of the neurolaryngology working group of the European Laryngological Society [18].

Regarding the first BTX injection, both adductor LD and vocal tremor were treated with 1.25 units bilaterally in the TA muscle. Subsequent injections were performed after symptoms recurrence and dosages were titrated in accordance with patients’ subjective symptomatic improvement and side effects.

### Statistics

A descriptive analysis of patient’s characteristics was performed taking into consideration absolute and relative frequencies for categorical variables, mean and standard deviation for normally distributed continuous variables and median and interquartile range for non-normally distributed continuous variables. Normality of continuous variables was assessed with the Kolmogorov-Smirnov Test. Evaluation of BTX treatment results was performed with the Paired Sample T-Test for parametric variables or the Wilcoxon Signed-Rank Test for non-parametric variables. Comparisons between groups were performed with Chi-square Test or Fischer’s Exact Test for categorical variables, Student’s T Test for normally distributed continuous variables and Mann-Whitney U Test for non-normally distributed continuous variables. All statistical analysis was made with the software IBM^®^ SPSS^®^ Statistics version 27 and associations were considered significant when *p* < 0.05.

This study was approved by the Ethics Committee of Hospital Universitario La Paz with the number 2024.147.

## Results

### Clinical characteristics

A total of 77 patients were included, where 44 had LD and 33 had VT. Clinical characteristics of LD and VT can be found in Table 1. Patients with VT had a higher age at diagnosis (69.8 ± 10.1 years) compared to LD (56.1 ± 16.7) (*p* < 0.001) while their symptom durations in months were similar (*p* = 0.821). There was also a higher female prevalence in the VT group (81.8%) in comparison to LD (54.5%) (*p* = 0.015).

**Table 1:** Clinical characteristics of patients with LD and VT

	LD (*n*=44)	VT (*n*=33)	*p*
Age (mean ± SD)	56.1 ± 16.7	69.8 ± 10.1	<0.001
Symptom Durations in Months (median ± interquartile range)	33 ± 84	24 ± 60	0.821
Female (%)	54.5%	81.8%	0.015
Adductor Dystonia (%)	100%		
Horizontal Tremor (%)		42.4%	
Mixed Tremor (%)		57.6%	
Adductor Dystonic Tremor (%)		66.6%	
Number of injections ≥ 2 (%)	68.2%	72.7%	0.802
**Diagnosis**			
Focal Laryngeal Disease (%)	79.5%	45.4%	
Meige Syndrome (%)	18.2%		
Generalized Dystonia (%)	2.3%		
Essential Tremor (%)		48.5%	
Parkinson’s Disease (%)		3%	
Stroke (%)		3%	

LD– Laryngeal Dystonia; SD– Standard Deviation; VT– Vocal Tremor

LD was predominantly a focal dystonia in 79.5% of the cases and was associated with more generalized neurological disorders in 20.5%. It was as an adductor type dystonia in all cases. VT was a focal pathology in 45.4% of patients and was associated with more generalized neurological disorders in 54.6%, with a majority having the diagnosis of essential tremor (48.5%). VT exhibited a horizontal tremor pattern in 42.4% of cases, a mixed pattern in 57.6%, and there were no cases of isolated vertical tremor. In the VT group, 66.6% of the patients had a dystonic tremor with an associated adductor type dystonia.

Pre-treatment parameters are shown in Table 2. There were no differences between groups regarding VHI-10 scores, grade of dysphonia on the VAS or MPT (*p* > 0.05). In the perceptual analysis, there were no differences in every parameter of the GRBAS-I scale with the exception of strain which was significantly higher in LD when compared to VT (*p* < 0.001).

**Table 2:** Values of the VHI-10, grade of dysphonia in the VAS, GRBAS-I scale and MPT at the first otorhinolaryngologic visit (Pre-treatment)

Pre-Treatment	LD (*n*=44)	VT (*n*=33)	*p*
VHI-10 (mean)	26.9	25.8	0.502
Grade of Dysphonia on the VAS (mean)	2.5	3.5	0.096
G (median)	2	2	0.664
R (median)	0	0	0.083
B (median)	0	0	0.119
A (median)	0	0	0.856
S (median)	2	1	<0.001
I (median)	2	2	0.637
MPT in seconds (mean)	16.7	15.0	0.436

A– Asthenia; B– Breathiness; G– Grade; I– Instability; LD– Laryngeal Dystonia; MPT– Maximum phonation time; R - Roughness; S - Strain; VAS– Visual Analogue scale; VHI-10– Voice Handicap Index– 10; VT– Vocal Tremor

### Results after treatment with BTX injection

**Fig. 1:**
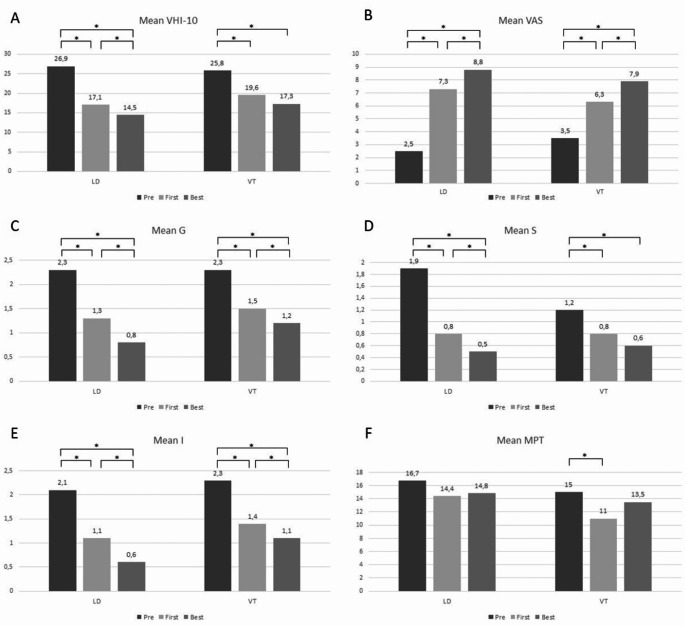
Results of treatments with BTX injection. The following variables were evaluated: A - Voice Handicap Index - 10; B - Grade of dysphonia in the Visual Analogic Scale; C - Grade in the GRAS-I scale; D - Strain in the GRAS-I scale; E - Instability in the GRAS-I scale; F - Maximum Phonation Time.Best– Outcomes 1 month following the injection that yielded the best response; First– Outcomes 1 month following the first BTX injection; LD - Laryngeal Dystonia; Pre– Outcomes at the first otorhinolaryngologic visit, before treatments; VT - Vocal Tremor * - *p* < 0,05

The results of treatments with BTX injections are presented in Fig. 1A-F. In the LD group, patients received an average of 6.0 BTX injections, while in the VT group, the average was 4.3 treatments, with no significant differences between the two groups (*p* = 0.155). Furthermore, 40.9% of patients with LD and 54.5% with VT decided to stop treatment during the follow-up (*p* = 0.258).

The first BTX injection was administered using the doses that were previously described in the methods section. For patients that were submitted to more than two injections (30 patients with LD and 24 with VT), the dose of BTX injection that yielded the best response was similar between both groups (*p* = 0.516), with an average of 4,17U for the LD group and 4,95U for the VT group. The interval between injections was also similar between both groups (*p* = 0.180), with 5.9 months for the LD group and 6.7 months for the VT group.

After the first BTX injection, patients with LD demonstrated a significant improvement in the VHI-10 score, grade of dysphonia on the VAS, and G, S, and I scores from the GRBAS-I scale (*p* < 0.005). However, there was no significant change in MPT. As the treatment continued, after the injection that yielded the best response, patients with LD showed a further significant improvement in the VHI-10 score, grade of dysphonia on the VAS, and G, S, and I scores from the GRBAS-I scale (*p* < 0.05), with no significant change on the MPT.

In the VT group, following the first BTX injection, patients exhibited a significant improvement in the VHI-10 score, grade of dysphonia on the VAS and G, S, and I scores from the GRBAS-I scale (*p* < 0.05), with a concomitant significant reduction in the MPT (*p* < 0.05). After the injection that yielded the best response, there was a further improvement in the grade of dysphonia on the VAS and G and I scores from the GRBAS-I scale (*p* < 0.05), with no significant variation on the VHI-10 score, S or MPT (*p* > 0.05).

Mean outcome variations following the first and the best BTX injection, as compared to the values at the first otorhinolaryngologic visit (pre-treatment), are presented in Table 3. After the first BTX injection, the LD group presented a higher improvement for the grade of dysphonia in the VAS (*p* = 0.010), and S (*p* = 0.006) in comparison to VT. Furthermore, the LD group showed a non-significantly higher improvement in the VHI-10 score (*p* = 0.072), G (*p* = 0.076) and I (*p* = 0.115) in comparison to VT.

**Table 3:** Mean outcomes variations following the first and the best BTX injection, as compared to the values at the first otorhinolaryngologic visit (pre-treatment)

Variation after 1st BTX Injection	LD (*n*=44)	VT (*n*=33)	*p*
VHI-10	-10.3	-6.1	0.072
Grade of Dysphonia on the VAS	4.8	2.7	0.007
G	-1.1	-0.8	0.076
S	-1.1	-0.7	0.006
I	-1.1	-0.8	0.115
**Variation after Best BTX Injection**	**LD** (*n*=30)	**VT** (*n*=24)	**p**
VHI-10	-13.2	-8.9	0.134
Grade of Dysphonia on the VAS	6.3	4.5	0.064
G	-1.6	-1	0.010
S	-1.4	-0.6	0.008
I	-1.6	-1.1	0.038

All are represented in means; G– grade; I– Instability; LD– Laryngeal Dystonia; S– Strain; VAS– Visual Analogue Scale; VHI-10; Voice Handicap Index– 10; VT– Vocal Tremor

As the treatment continued, after the injection that yielded the best result, the LD group has shown a higher improvement in G (*p* = 0.010), S (*p* = 0.008) and I (*p* = 0.038) in comparison to VT. Additionally, the LD group had a non-significantly higher improvement in the VHI-10 score (*p* = 0.134) and the grade of dysphonia on the VAS (*p* = 0.064) in comparison to VT.

## Discussion

The main objective of this study was to assess and compare the results of BTX injection treatment between patients with LD and VT. We have found that BTX treatments have improved significantly the VHI-10, grade of dysphonia on the VAS and G, S and I from the GRBAS-I scale in both patients with LD and VT. Furthermore, there was no significant alteration in the MPT except for patients with VT after the first injection. When we have compared treatment responses between patients with LD and VT, we have found that patients with LD seem to have a better response following treatment with BTX.

Many treatments have been proposed for LD. Truthfully, many authors have tried to treat it with oral medications, such as baclofen or benzodiazepines, with limited success [11]. Aside from some rare cases of dystonia responsive to levodopa, there are no drugs with proven efficacy to treat LD [19]. Since *Blitzer* described the use of BTX injection in 1986 [20], it has been considered by most authors the mainstay of treatment [9]. In fact, many previous studies have shown an improvement in the VHI, subjective self-assessed grade of dysphonia, mean fundamental frequency, perturbation and spectrographic analysis, jitter, voice break factor and mean airflow with no variation in the maximum phonation time, following BTX injection [21–23]. In accordance with these results, patients with LD in this study have shown an improvement in the VHI-10, grade of dysphonia in the VAS, G, S and I in the GRBAS-I scale after treatment with BTX injection with a further improvement in all these variables with further injections. As treatment with BTX injection is temporary, and repetitive injections are required in order to control LD symptoms, some authors use more permanent treatments such as type II thyroplasty [24], laser mioneurectomy [25] and denervation-reinnervation procedures [26]. Even though these procedures seem to be effective in the long-term, they are more invasive and irreversible. We have been doing BTX injections in our institution for over a decade, with good results, and our patients usually prefer a less invasive and reversible treatment, even with the need of repetitive procedures. Furthermore, as *Sataloff* stated, a cure for LD may occur in our lifetime and while BTX treatment would not interfere with a patient’s ability to receive curative therapy, surgery may lead to irreversible changes that can interfere with it [27].

In our practice, BTX injections are performed with electromyographic control. We believe that with electromyographic controlled injections are more accurate than with transoral or transnasal injections and that injection accuracy is important for treatment success. On the other hand, in a randomized trial there were no differences in outcomes between EMG and fiberoptic guidance injections in the treatment of adductor spasmodic dysphonia in experienced hands [28]. Thus, in experienced hands, fiberoptic guided BTX injections may be a viable alternative in centers without access to laryngeal EMG, but in cases of treatment failure or inexperience with this technique, we believe that patients with LD should be referred to centers with access to laryngeal EMG for BTX treatment.

VT is treated in most cases in accordance to the underlying associated pathology such as propranolol or primidone for essential tremor and levodopa for Parkinson’s disease [12]. However, since these medications don’t seem to be as effective to treat laryngeal tremor, many authors advocate the use of BTX injections to treat VT [14]. In fact, many studies have shown that treatment with BTX injection improves the VHI-10, acoustic and perceptual measurements in patients with VT [29–31]. In accordance, we have found that BTX injection has led to an improvement int the VHI-10 score, grade of dysphonia on the VAS and G, S, and I scores from the GRBAS-I scale, with a further improvement of the VAS, G and I with the continuation of the treatment. In all patients from our cohort, BTX injection was performed bilaterally in the TA muscle. Some recent studies point for an improvement in symptomatic control in patients with mixed or vertical VT when a concomitant strap muscle injection is performed [32]. Thus, with a careful selection, the concomitant injection in the strap muscle of patients with VT may further improve our results in the VT group, but since we have only started to do so recently and haven’t included patients with strap muscle injection in this study, we don’t have yet sufficient data to support this hypothesis.

Some patients with VT associated with Parkinson’s disease or essential tremor, may also present with hypophonia and glottic insufficiency, which has been treated by some authors with injection augmentation [33, 34]. A cross-over study compared the success of essential vocal tremor treatment with BTX and injection augmentation and found that there is no clear advantage of injection augmentation in comparison to BTX and suggest that a trial for augmentation should be reserved for patients whose symptomatic control with BTX is poor enough to discontinue the treatment [35]. We believe that the concomitant presence of hypophonia and glottic insufficiency in patients with Parkinson’s disease and essential tremor may justify a significant decrease in the MPT after the first BTX injection in patients with VT. In fact, BTX injections result in a temporary paresis of the TA muscle that could further increase the glottic insufficiency.

Previous studies seem to show a superior improvement after BTX injections in patients with dystonic tremor in comparison to VT [31, 36]. In this study we have included patients with dystonic tremor in the VT group since treatment results were more similar to VT in comparison to LD and in order to increase the sample of the VT group. While we understand that this may have led to a bias, both dystonic tremor and isolated VT patients have shown a significant improvement in the VHI-10, grade of dysphonia in VAS and G and I in the GRBAS-I scale (results not reported). Furthermore, there were no significant differences in outcome variations following treatment of dystonic tremor and isolated VT, even though these results might be biased due to a small sample size when we separate these two groups (results not reported). It would be of particular interest to do future studies with higher samples (for example, multicentric studies) that could compare the results with BTX treatment between LD, dystonic tremor and isolated VT.

To our knowledge, there are no previous studies comparing treatment responses between LD and VT following BTX injection. We have found patients with LD had a significant higher improvement in the grade of dysphonia on the VAS and S and a non-significant but higher improvement in the VHI-10 scale, G and I after the first injection. Furthermore, with the continuation of treatment, patients with LD have shown a significant higher improvement in the G, S and I and a non-significant higher improvement in the VHI-10 scale and VAS.

It was previously found that patients with LD seem to need relatively higher doses of BTX (6.8 U) in comparison to VT (5.02 U) [37]. On the other hand, we didn’t find any significant difference in the BTX dose that yielded the best response neither in the treatment interval between LD (4.17 U; 5.9 months) or VT (4.95 U; 6.7 months). This difference may be explained by a smaller sample size in our study or differences in exclusion criteria, since *Orbelo* et al. have excluded patients with more generalized neurological disorders [37].

This study has some limitations. Firstly, it is an observational retrospective study with possible bias due to lack of information on clinical data. Furthermore, laryngeal movement disorders are rare and a smaller sampler size may limit statistical differences. Thus, to increase sample size, we have included patients with dystonic tremor in the VT group as we have discussed previously. Moreover, we haven’t excluded patients with more generalized neurological disorders, since, in our experience, they seem to have similar benefit with BTX treatment and they represent an important percentage of our practice. Lastly, the differential diagnosis between LD, VT and dystonic tremor is difficult and requires a high level of experience. In order to reduce misdiagnosis to a minimum, we have used a detailed diagnosis protocol that was previously described by our group [3].

## Conclusions

To conclude, BTX injection was successful in improving the VHI-10, grade of dysphonia on the VAS and G, S and I in the GRBAS-I scale for both DT and VT. LD seems to have a better response to BTX in comparison to VT.
